# Prevention and management of human cytomegalovirus in pediatric HSCT recipients: A review

**DOI:** 10.3389/fped.2022.1039938

**Published:** 2022-11-23

**Authors:** Lisa Hiskey, Theresa Madigan, Elizabeth H. Ristagno, Raymund R. Razonable, Asmaa Ferdjallah

**Affiliations:** ^1^ Division of Pediatric Infectious Diseases, Department of Pediatrics and Adolescent Medicine, Mayo Clinic, Rochester, MN, United States; ^2^ Division of Public Health, Infectious Diseases and Occupational Medicine, Mayo Clinic, Rochester, MN, United States; ^3^ Division of Pediatric Hematology and Oncology, Department of Pediatrics and Adolescent Medicine, Mayo Clinic, Rochester, MN, United States

**Keywords:** cytomegalovirus, pediatrics, hematopoietic (stem) cell transplantation (HSCT), immunosuppression, infection, herpesviruses

## Abstract

Cytomegalovirus (CMV), like other herpesviruses, has the unique ability to establish latent infection with subsequent reactivation during periods of stress and immunosuppression. Herpesviruses cause potentially devastating disease, particularly in hematopoietic stem cell transplant (HSCT) recipients. CMV is especially of concern in HSCT recipients given the high community seroprevalence, high risk of reactivation and high risk of transmission from HSCT donors to recipients causing primary infection after transplantation. The risk of CMV infection and severity of CMV disease varies depending on the underlying disease of the HSCT recipient, donor and recipient CMV status prior to HSCT, type of conditioning therapy in preparation for HSCT, allogeneic versus autologous HSCT, donor graft source, timing of infection in relation to HSCT, and other patient comorbidities. Different strategies exist for prevention (e.g., preemptive therapy vs. universal prophylaxis) as well as management of CMV disease (e.g., antiviral therapy, augmenting immune reconstitution, cytotoxic T-cell therapy). The purpose of this narrative review is to discuss diagnosis, prevention, and management of CMV infection and disease at different stages of HSCT, including key points illustrated through presentations of complex cases and difficult clinical scenarios. Traditional and novel strategies for CMV management will be discussed in the context of these unique clinical cases.

## Introduction

Hematopoietic stem cell transplant (HSCT) is a curative therapy for several diseases in pediatric patients, including hematologic malignancies, primary immune deficiencies, myelodysplastic syndrome, congenital metabolic disorders, and hemoglobinopathies (1, 2). More than 12,000 HSCTs were performed in children <18 years of age in the United States between 2016 and 2020 (3). HSCT recipients have severely compromised immune systems (due to their underlying disease and secondary to HSCT conditioning), increasing their risk for bacterial, fungal, viral, and parasitic infections. These infections can be derived from donors, environmental sources or *via* reactivation of endogenous latent infections (4, 5).

Among viral infections, herpesviruses, particularly cytomegalovirus (CMV), present significant concerns for infection in HSCT patients. CMV has a high community seroprevalence with approximately 50% of people in the United States seropositive, with variations depending on age, geography, and socioeconomic status (6, 7). Many patients will therefore enter HSCT with an established latent CMV infection (as indicated by a positive pre-transplant CMV IgG serology). In addition, CMV can also be transmitted to HSCT recipients from the donor. This may result in devastating and possibly fatal disease with the potential to precipitate several indirect outcomes such as graft-vs.-host-disease (GVHD), autoimmunity, malignancy, and increased risk of other opportunistic infections (4, 8, 9).

## Pre-transplant evaluation

Among many other predisposing factors, the risk of CMV infection and disease is highly dependent on the combination of recipient and donor CMV serostatus. HSCT recipients are at highest risk of CMV infection when the recipient is CMV seropositive and the donor is CMV seronegative pre-transplant (9). In this case, the recipient will be at high risk for endogenous CMV reactivation during immune suppression following HSCT, at a time when the cell-mediated immune system is suppressed from the conditioning regimen for HSCT. Eventually, there will be a gradual development or reconstitution of CMV-specific T-cell immunity, but this may take time (10). Fifty percent of patients develop detectable CMV cytotoxic T-cell (CTL) response by 3 months after allogeneic HSCT, and reconstitution of CMV-specific CD4+ and CD8+ T-cells has been shown to be a good indicator of absolute CD4+ and CD8+ T-cell numbers (11, 12). Failure to produce CMV-specific immunity by 3 months post-HSCT has been shown to be significantly associated with late CMV reactivation and increased mortality (13). Among CMV seropositive patients, approximately 80% will experience CMV reactivation after allogeneic HSCT in the absence of CMV prophylaxis (14).

When interpreting serologies, it is important to consider pre-transplant receipt of intravenous immunoglobulin (IVIG) or blood products within the preceding 8–11 months, as these treatments are common among pre-HSCT recipients and can lead to false-positive serologies (15, 16). Care should also be taken when interpreting CMV serologies in infants ≤12 months of age, given influence of transplacental maternal antibody (17, 18).

Providers should also consider the graft source. Matched unrelated, mismatched and HLA-haploidentical transplants have an increased risk of CMV infection compared to matched related transplants, possibly secondary to greater immune suppression (9, 19–21). Receipt of T-cell depleted or umbilical cord allografts (which are deficient in CMV-specific T-cells) are particularly associated with a very high risk of CMV infection.

It is also important to consider if the patient is currently breastfeeding, as CMV can be transmitted *via* breast milk. Up to 96% of CMV seropositive breastfeeding mothers develop CMV reactivation at some time during lactation (22). Amongst patients with severe combined immune deficiency breastfed by CMV seropositive mothers, there is a 5%–6% CMV transmission rate (23). For mothers of infants undergoing evaluation for HSCT, consideration can be given to testing for CMV antibodies. Some advocate for CMV seropositive mothers to refrain from breastfeeding due to the risk of CMV transmission and the potential for devastating outcomes (24).

## CMV prevention strategies

There are two main strategies for CMV disease prevention following HSCT: preemptive therapy and universal prophylaxis. The overall risk of CMV disease is considered in determining the appropriate preventive approach.

Preemptive therapy involves instituting serial CMV monitoring and beginning CMV antiviral therapy at a pre-defined threshold of viral load (25). This approach is more likely to be considered in patients deemed to be at a lower risk of CMV reactivation. Preemptive therapy involves once weekly CMV quantitative polymerase chain reaction (PCR) monitoring until day +100 with initiation of CMV-active antivirals if CMV PCR becomes positive or rises above a certain level. Though some thresholds have been suggested, there is no widely-accepted universal viral load threshold at which to initiate therapy; the decision to initiate therapy should be determined by each treatment center based on the assay used, patient risk factors (e.g., donor/recipient CMV serostatus, overall state of immune suppression), and the rate of rise of viral load (7, 26).

Universal prophylaxis is the strategy of administering anti-CMV drug prophylaxis (Table 1) to at-risk recipients prior to the development of CMV viremia at a pre-defined time point after transplant. Universal prophylaxis is often pursued in patients at higher risk such as recent primary CMV infection immediately prior to transplant, CMV-seropositive patients receiving a graft from seronegative donors, those who receive T-cell depleting therapies (e.g., alemtuzumab or antithymocyte globulin) and recipients of T-cell depleted, HLA-mismatched, haploidentical or umbilical cord blood allografts (36). Potential adverse effects of antiviral medications are an important consideration with use of universal prophylaxis. Ganciclovir and valganciclovir have the undesirable side effect of myelosuppression, which can delay or reverse neutrophil engraftment. The resulting prolonged lymphopenia and/or neutropenia places the patient at risk for other opportunistic bacterial and fungal infections (37, 38). Foscarnet and cidofovir could also be considered and may be preferred due to less bone marrow toxicity. However, these medications can lead to renal toxicity and/or electrolyte abnormalities. Letermovir has been Food and Drug Administration (FDA) approved for CMV prophylaxis in adult HSCT recipients aged 18 years or older, though has not yet received approval for use in children (27). Despite this, several centers have begun using letermovir in pediatric HSCT patients and have reported promising outcomes (39–42). Maribavir is the newest antiviral to have received FDA approval. This medication is only approved for the treatment of refractory/resistant CMV infection or disease in patients 12 years of age and older and weighing ≥35 kg, and has not been approved for prophylaxis (28).

**Table 1:** Antiviral prophylaxis and treatment in pediatric HSCT recipients (27–35).

Medication	Prophylaxis	Prophylaxis dose^a^	Treatment	Treatment dose^a^
Ganciclovir	Y	5 mg/kg/dose IV q24 h	Y	5 mg/kg/dose IV q12 h
Valganciclovir	Y	7 × BSA^b^ × CrCl^c^ PO q24 h (max 900 mg/day)	Y	7 × BSA^b^ × CrCl^c^ PO q12 h (max: 900 mg/dose)
Foscarnet^d^	Y	60 mg/kg/dose IV q12 h for 7 days then 90–120 mg/kg/dose qDay	Y	60 mg/kg/dose IV q8 h; Maintenance: 90 mg/kg qDay
Cidofovir^e^	Y^f^	5 mg/kg/dose qWeek × 2 weeks then 5 mg/kg/dose every other week	Y	5 mg/kg/dose qWeek × 2 weeks then 5 mg/kg/dose every other week
Letermovir (≥18 years)	Y	480 mg PO IV q24 h	N	NA
Maribavir (≥12 years and ≥35 kg)	N	NA	Y	400 mg PO BID

^a^ Dosing given is for patients with normal renal function.

^b^ BSA, body surface area.

^c^ CrCl, creatinine clearance, using modified Schwartz formula which bases k constant on age.

^d^ IV hydration should be given as 10–20 ml/kg (max 1,000 ml) prior to initial infusion and then 10–20 ml/kg (max 1,000 ml) given concurrently with subsequent doses.

^e^ Should be given with probenecid (25–40 mg/kg/dose (max 2,000mg) PO 3 h prior to cidofovir and 10–20 mg/kg/dose (max 1,000 mg) 2–3 h and 8–9 h after cidofovir) as well as IV hydration (10–20 ml/kg pre- and post-cidofovir OR increase maintenance IVF by 1.5–2×).

^f^ Less commonly used due to availability of other agents with more favorable side effect profiles.

Another proposed prevention strategy is pre-transplant ganciclovir or valganciclovir. With this strategy, CMV seropositive patients receive ganciclovir or valganciclovir at the start of conditioning and through day −2. Patients are subsequently followed by preemptive therapy as noted above. Research has shown lower rates of CMV reactivation amongst patients receiving pre-transplant ganciclovir, with incidence of reactivation comparable to patients receiving letermovir (43). One study showed earlier time to reactivation amongst patients who did not receive pre-transplant valganciclovir, though noted no overall impact on rate of CMV reactivation or survival at 100 days (44).

Blood transfusions carry an additional risk of CMV transmission. Transfusion-associated CMV infection occurs due to reactivation of latent CMV infection in transfused monocytes (45), although the risk is exceedingly small with the use of leukoreduced blood products (46). Therefore, only CMV-negative or leukocyte-reduced blood products should be administered to patients in whom HSCT is anticipated or planned (47).

CMV hyperimmune globulin (CMVIG) is not recommended for routine use for prophylaxis in pediatric HSCT recipients. While some research has indicated that receipt of IVIG may decrease risk of CMV infection or disease, particularly in the first year after transplant, other studies have indicated no benefit beyond what is provided by antiviral drugs (48–50).

Despite a decades-long effort to develop a CMV vaccine, there is no vaccine available for clinical use. Research is ongoing regarding vaccinations to boost CMV immunity in high-risk patients. There are several vaccines under investigation, including clinical trials in pediatric patients (51, 52).

## Diagnosis of CMV infection and disease

In this section, we will discuss general diagnostic principles. Further details on diagnosis of specific disease manifestations are discussed in the relevant case presentations.

When CMV is detected in a clinical sample, it should then be determined if the patient is experiencing CMV infection or CMV disease. CMV infection is defined as the presence of CMV replication in tissue, blood, or other bodily fluids regardless of symptoms. CMV disease is the presence of CMV infection in the setting of attributable symptoms (e.g., fever, hypoxia, or diarrhea). CMV disease is generally divided into CMV syndrome (a term used only in solid organ transplantation) or CMV end-organ disease. CMV syndrome often manifests with constitutional symptoms of fever and malaise as well as laboratory findings of atypical lymphocytosis, leukopenia, neutropenia, thrombocytopenia and/or elevated hepatic transaminases; this terminology is generally not used in HSCT because of the common occurrence of the signs and symptoms (e.g., leukopenia, thrombocytopenia) even in the absence of active CMV replication. CMV end-organ disease presents with symptoms in the affected organ, such as abdominal pain or diarrhea in gastrointestinal disease or hypoxia, dyspnea, and new pulmonary infiltrates in pneumonia (7, 14).

Nucleic acid amplification testing (NAT) is the preferred method of diagnosis of CMV infection. This testing most commonly uses PCR to detect viral DNA (or, less commonly, RNA). Detection of RNA is a more specific marker for viral replication (but it is a less sensitive target), while presence of DNA does not necessarily reflect active viral replication (7, 53–55). There is currently no commercial assay available for CMV RNA. When NAT testing is performed, quantitative methods should be used. Quantitative methods allow differentiation between detection of latent virus (e.g., low-level DNA-emia) vs. active replication (such as with high or rising viral load) and allow for monitoring of change in viral load over time. The change in viral load is important to measure treatment response, progression of viremia and risk of CMV disease (7). Research has indicated that a higher initial viral load as well as a higher logarithmic rate of rise in viral load are both risk factors for development of CMV disease (56).

Histopathology is the gold standard for definitive diagnosis of end-organ CMV disease (7, 57). Samples can be collected from the source tissue of interest, such as the intestine or lung. Hematoxylin and eosin preparations as well as immunohistochemical stains are performed and the samples are evaluated for CMV viral inclusions (58). The exception to this is CMV retinitis, which is diagnosed primarily through classic ophthalmologic examination findings, with PCR of vitreous fluid used only at times to confirm the diagnosis, particularly in atypical cases (9, 59, 60). It should be noted that obtaining samples for confirmative histopathology review may not always be feasible given the inherent invasive nature of this testing. Often, HSCT patients have thrombocytopenia that limits the performance of invasive procedures.

Other methods of testing, including pp65 antigen testing (detection of CMV antigen on peripheral blood leukocytes) and conventional or shell vial viral culture, have largely fallen out of favor in the era of molecular assays. Viral culture, though highly specific for diagnosis of CMV infection, has poor sensitivity and takes longer to result (61, 62). CMV pp65 antigenemia on the other hand is labor intensive and lacks standardization (7).

## General management strategies

Management of CMV infection and disease in HSCT patients requires a multidisciplinary approach involving the infectious diseases specialist, stem cell transplant physician, pharmacist, and other providers. Immunosuppression should be reduced as a first step, as rapidly as possible (7). In allogeneic HSCT recipients, this may mean a rapid wean and discontinuation of tacrolimus, sirolimus, mycophenolic acid or other prophylactic drugs against GVHD. In cases of asymptomatic, low-grade CMV viremia, this may be the only intervention necessary to control infection.

However, in some HSCT patients, particularly those with active GVHD, reduction of immune suppression may not always be feasible. Antiviral therapy is often necessary for management of CMV infection and disease in these patients.

First-line antiviral agents are intravenous (IV) ganciclovir and oral (PO) valganciclovir. As noted previously, these agents are myelosuppressive. IV ganciclovir is recommended for initial management in those with severe disease, very high viral load, and those with concerns regarding absorption. PO valganciclovir is a reasonable option in mild-moderate disease when the patient can reliably take oral medication. Valganciclovir is also used as oral step-down therapy in patients with CMV disease who have demonstrated good clinical and virologic response to initial IV ganciclovir treatment (7, 36). Doses of ganciclovir and valganciclovir are noted in Table 1.

**Figure 1:**
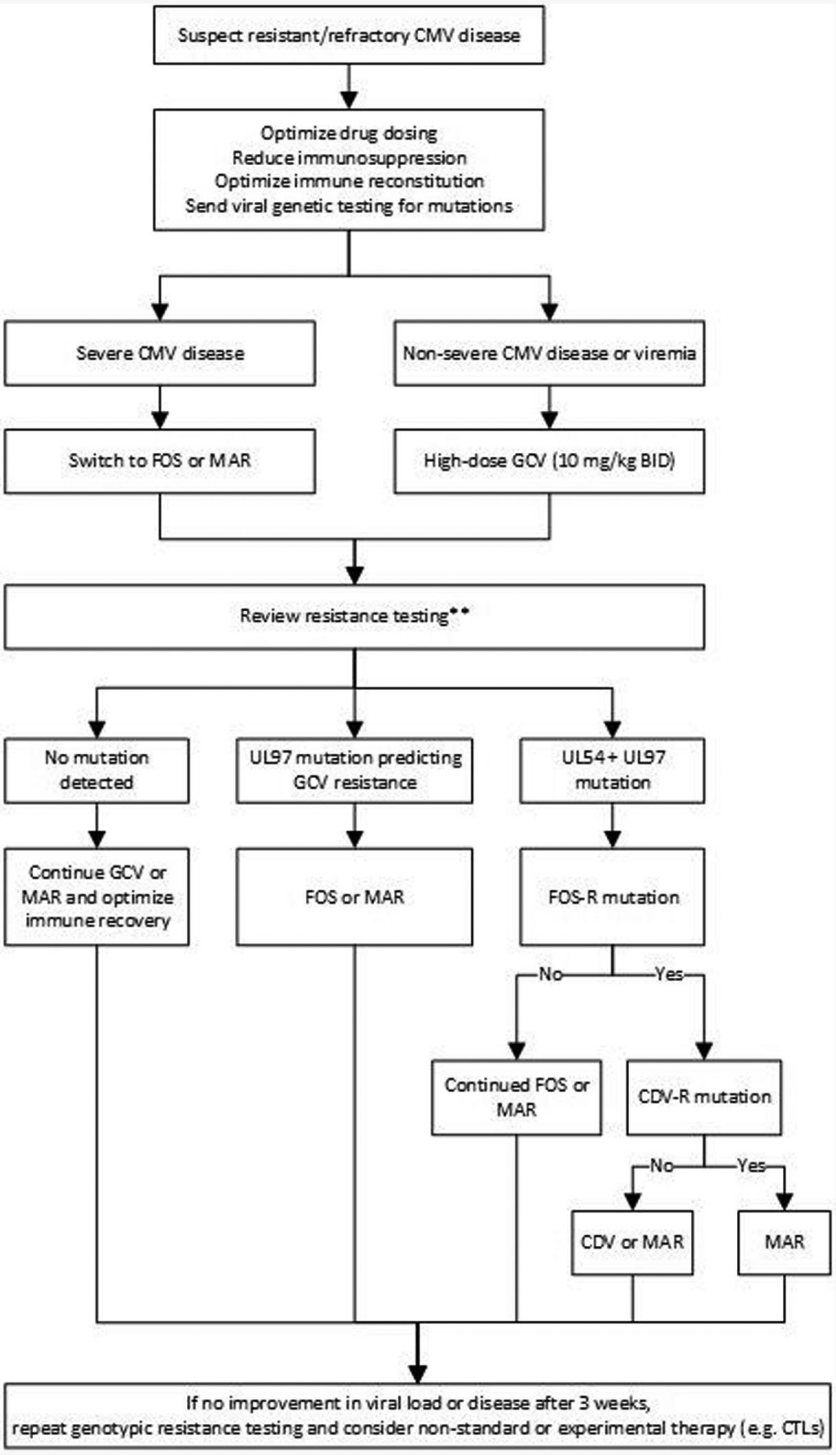
Proposed treatment algorithm for refractory/resistant CMV infection or disease (7, 9, 63, 64). GCV, ganciclovir; FOS, foscarnet; MAR, maribavir; CDV, cidofovir. **In the rare instance of UL54 mutation that predicts FOS-R alone (GCV-S) and without UL97 mutation, resume GCV.

Other antiviral medications include foscarnet and cidofovir. Both medications are only available in IV form, and both are nephrotoxic. In some centers, foscarnet is the preferred drug for CMV treatment in the pre-engraftment period given concerns of bone marrow toxicity with ganciclovir and valganciclovir (Table 1). Additionally, both medications can be used for treatment of refractory or resistant CMV infection or disease (Figure 1) (7).

Maribavir is a CMV antiviral agent that was approved in November 2021 for treatment of refractory and/or resistant CMV infection and disease in adults and children aged 12 years or older and weighing at least 35 kg (28). As noted previously, letermovir is approved for CMV prophylaxis, though is not approved for treatment of CMV infection or disease (Table 1) (27). There are case reports of letermovir use as salvage therapy in refractory/resistant CMV infection, however, there is concern for low threshold for resistance following exposure to letermovir (65, 66).

CMV antiviral therapy should be continued until symptomatic resolution and viral clearance, and all patients should receive at least 2 weeks of therapy. Depending on the sensitivity of the assay used, viral clearance may be defined as undetectable viral load for 1–2 weeks (7). The role of secondary antiviral prophylaxis is debated but may be considered for HSCT patients with ongoing risk factors for recurrence of CMV infection. If secondary antiviral prophylaxis is not provided, HSCT patients should undergo weekly CMV surveillance to monitor for recurrence or relapse (47).

A more recent investigational therapy is the utilization of CMV-specific cytotoxic T lymphocytes (CTLs). CTLs are produced by using CMV antigen peptides to induce CMV-specific T-cells in donor blood (67). There are limited studies on the use of CTLs for treatment of CMV infection in children, though available data suggest this could be a safe and effective therapy for treatment in pediatric HSCT recipients (68, 69). Availability of this therapy is currently limited to clinical trials.

CMVIG is another available therapeutic option in addition to an antiviral drug. CMVIG is a pooled plasma product containing a high titer of anti-CMV antibody (70). CMVIG has been investigated as salvage therapy in adults with CMV infection post allogeneic HSCT (71, 72). While this therapy has been well-tolerated, the benefit has not been proven. CMVIG has also been used as salvage therapy in pediatric populations, though pediatric-centered research is lacking. Additionally, intrathecal CMVIG has been suggested as a potential adjunctive treatment for CMV encephalitis. With few anecdotal cases in adults showing mixed results, this is not regarded as a preferred strategy (73, 74).

Non-CMV-specific high-dose IVIG has historically been used in management of CMV disease, particularly in treatment of CMV pneumonia (75). However, recent research has failed to clearly support the role of IVIG these patients (75–77). Current pediatric HSCT guidelines recommend IVIG therapy only in cases of hypogammaglobulinemia (78, 79).

## Clinical case examples

### Case presentation 1: CMV DNAemia pre-transplant

A 6-month-old boy with Wiskott-Aldrich Syndrome was admitted for conditioning in preparation for a matched unrelated donor bone marrow transplant (MUD HSCT) (CMV D−/R+). Pre-transplant antimicrobial prophylaxis included daily trimethoprim-sulfamethoxazole, amoxicillin, and monthly IVIG. Pre-transplant infectious diseases work-up demonstrated positive CMV, Epstein-Barr (EBV) and herpes simplex virus (HSV) IgG though interpretation of these results was complicated by recent receipt of IVIG as well as possible maternal antibodies. A CMV viral load was obtained one day prior to transplant and was noted to be 5,000 IU/ml. The patient had no evidence of CMV disease, including retinitis. He received busulfan and cyclophosphamide for conditioning and underwent BMT as planned. He subsequently received GVHD prophylaxis with daily tacrolimus 0.6 mg BID, a single dose of alemtuzumab 3 mg and mini methotrexate 1.75 mg on days +1, +3, +6 and +11.

Due to CMV viremia, he was started on foscarnet 90 mg/kg/dose IV q12 h on day +1 for treatment, in order to avoid bone marrow toxicity associated with ganciclovir pre-engraftment. On day +11, CMVIG was initiated in addition to antiviral therapy. At the end of 2 weeks of therapy, the CMV viral load demonstrated a nearly 1-log increase. Though resistance testing through next-generation sequencing did not demonstrate any drug resistance-conferring mutations, there was concern for foscarnet resistance and the patient was switched to induction dosing ganciclovir of 5 mg/kg IV q12 h which was subsequently increased to 10 mg/kg IV q12 h due to a further rising viral load. The viral load then demonstrated a slow improvement and ganciclovir dosing was decreased to 7.5 mg/kg q12 h and then back to 5 mg/kg q12 h.

On day +31, approximately 1 month into antiviral therapy and shortly after the decrease in ganciclovir dosing to 5 mg/kg q12, the patient started to require supplemental oxygen. A computed tomography (CT) scan of the chest revealed diffuse ground-glass opacities in the setting of a rising viral load up to 110,000 IU/ml (5.04 log). The patient was transitioned back to foscarnet 60 mg/kg q8 h. A bronchoalveolar lavage (BAL) was concerning for diffuse alveolar hemorrhage (DAH). The CMV PCR on BAL fluid was positive, as would be expected with DAH in a patient with significant viremia. Cytology from the BAL fluid showed abnormal epithelial cells favored to be of a reactive/degenerative etiology with rare degenerative cells demonstrating staining suspicious for CMV. The patient required oxygen therapy for several days during this evaluation, though was quickly weaned to room air. It was felt that DAH was the primary contributor to the patient's respiratory symptoms, though CMV likely played a role, as well.

During this time, the patient's absolute lymphocyte count (ALC) remained profoundly low at <0.1 × 10^9^/L (normal 1.56–7.83 × 10^9^/L). This was potentially a combined consequence of recent HSCT with myeloablative conditioning, alemtuzumab, CMV infection, and the bone marrow suppressive effects of intermittent ganciclovir. Due to persistent lymphopenia likely contributing to the difficulty in controlling infection, CTL therapy was considered. However, given the recent receipt of alemtuzumab, a T-cell antibody, CTL therapy was initially deferred. The patient underwent plasmapheresis to remove alemtuzumab, with close monitoring of alemtuzumab levels. Once the alemtuzumab level was <0.15 μg/ml, the patient was referred to a nearby study center for CTL therapy and received 2 doses, given 3 weeks apart. The CMV viral load subsequently decreased and repeat resistance testing was again negative. The ALC demonstrated improvement to 1.71 × 10^9^/L. With an improved viral load and lymphocyte recovery, the patient was transitioned to valganciclovir 180 mg PO BID for home-going therapy.

#### Case discussion

This case highlights the unique challenges of managing CMV viremia before and during bone marrow transplant while awaiting immune reconstitution. Patients with CMV DNAemia at the time of transplant are among the highest risk patients for CMV infection and disease (9). This patient's course was complicated by delayed lymphocyte recovery, likely a result of the combination of his myeloablative conditioning regimen, GVHD prophylaxis with alemtuzumab, CMV-induced lymphopenia, and possible contribution of bone marrow suppression secondary to intermittent therapy with ganciclovir.

Also highlighted in this case is the patient's diagnosis of probable CMV pneumonia. He had bronchoscopy findings concerning for DAH with cytology suspicious for CMV. DAH is an uncommon complication of HSCT, typically occurring in the early post-transplant period. Additionally, DAH in HSCT patients is, by definition, non-infectious (80). However, CMV viremia with cytology suspicious for CMV was concerning for possible contribution of CMV to the patient's respiratory symptomatology.

While not the clear sole cause of this patient's symptoms, CMV pneumonia is a significant concern in immunocompromised patients. CMV pneumonia is one of the most severe manifestations of CMV infection in HSCT recipients and has a mortality rate of up to 50% even with treatment (81, 82). This diagnosis is made by a combination of new infiltrates on imaging and respiratory symptoms (e.g., tachypnea, dyspnea, hypoxia) in the setting of CMV detected in lung tissue or BAL fluid. The diagnosis is considered proven if CMV is documented in lung tissue by viral isolation, culture, histopathology, immunohistochemistry, or DNA hybridization techniques and probable if CMV is detected in BAL fluid by viral isolation, culture, or PCR (7).

### Case presentation 2: Resistant CMV

In continuation of case #1, the patient was noted to have a rising CMV viral load once again after 4 months of antiviral therapy [from 653 (2.81 log) to 3,310 IU/ml (3.52 log)]. He also had chronic diarrhea, which raised concern for CMV intestinal disease or gastrointestinal GVHD. A duodenal biopsy was obtained and demonstrated sparse inflammatory cells in the lamina propria with crypt apoptosis and negative CMV immunostain. While crypt apoptosis can be associated with GVHD, the patient was also noted to have *Clostridioides difficile* infection, which can also cause these findings (83, 84). The patient had no other symptoms suggestive of CMV disease, including respiratory compromise. Given prolonged exposure to ganciclovir, valganciclovir and foscarnet, resistance testing was performed, and showed a L595W mutation in the UL97 gene, predicting resistance to ganciclovir. Notably, although the patient's ALC had improved to around 1.0 × 10^9^/L, quantitative lymphocyte subsets revealed primarily (70%) CD19 cells with a CD4 count of 12 cells/mcl, CD8 count of 2 cells/mcl and CD3 count of 28 cells/mcl. T-cell receptor excision circles (TREC) analysis demonstrated pan-T-cell lymphopenia, consistent with poor T-cell reconstitution following HSCT. It was felt that lymphopenia was contributing significantly to the patient's ongoing CMV viremia. For therapy optimization, the patient was transitioned to foscarnet 60 mg/kg q8 h and received a third dose of CMV-specific CTLs.

After 2 months on foscarnet (and approx. 6 months post-transplant), the patient continued to have detectable viral load, rising again to a height of 7,900 IU/ml. He was re-admitted due to hematemesis. Ophthalmologic exam showed CMV retinitis with small intraretinal hemorrhages and small subretinal lesions amenable to monitoring. Repeat resistance testing revealed a A834P mutation of UL54 (with no UL97 mutation), predicting resistance to ganciclovir, foscarnet and cidofovir. His ALC had improved to 2.01 × 10^9^/L, though still with predominance (59%) of CD19 cells, and improved but persistently low CD4 count of 141 cells/mcl, CD8 count of 157 cells/mcl and CD3 count of 377 cells/mcl. Ganciclovir was restarted, in addition to foscarnet.

After approximately 1 week on dual antiviral therapy, the patient developed respiratory distress with hypoxia and increased work of breathing. A CT chest revealed bilateral opacities. The CMV viral load was 1,100 IU/ml. Due to concern for CMV pneumonia in the setting of multi-drug resistant CMV, ganciclovir was discontinued, and the patient was started on maribavir 400 mg/dose BID. CMVIG was continued weekly. Within 24 h of transition to maribavir, however, the patient developed worsening respiratory distress and required transfer to the pediatric intensive care unit. Bronchoscopy with BAL demonstrated normal lower airways. The BAL fluid analysis showed a total nucleated cell count of 1.8 and predominance of alveolar macrophages. Infectious diseases work-up on the BAL fluid showed negative bacterial, fungal and mycobacterial cultures, as well as negative aspergillus antigen and negative PCRs for *Pneumocystis jirovecii*, adenovirus, CMV, influenza and respiratory syncytial virus. An esophagogastroduodenoscopy showed esophageal ulcers; biopsy of the esophagus revealed reactive squamous esophageal mucosa with rare inflammatory infiltrate, no apoptotic bodies, no definitive GVHD, negative periodic acid-Schiff (PAS) stain and negative CMV immunostain.

CMV viral load remained elevated at 1,620 IU/ml 3 days after initiation of dual therapy with maribavir and foscarnet. The patient received a fourth dose of CTLs. The ophthalmologic exam remained stable. The patient unfortunately progressed to severe acute respiratory distress syndrome (ARDS) with refractory hypoxia, necessitating transition to extracorporeal membrane oxygenation (ECMO). Suspicion rose for an alternative etiology of ARDS, including DAH, idiopathic pneumonia syndrome (IPS) or cryptogenic organizing pneumonia. The patient was started on methylprednisolone. After approximately 2 weeks on maribavir, the CMV viral load showed improvement, decreasing to 550 IU/ml. However, given the patient's critical status, he was also started on letermovir 240 mg IV BID, leflunomide 5 mg PO q24 h and artesunate 3 mg/kg IV q24 h for additional CMV-active antiviral therapy. A lung biopsy revealed acute lung injury with predominant features of organizing diffuse alveolar damage and a component of necrotizing bronchiolitis. Immunohistochemical stains were initially negative for CMV, varicella zoster virus (VZV), adenovirus and HSV 1 and 2. With initial negative infectious work-up of lung biopsy, artesunate, leflunomide and letermovir were discontinued and the patient was started on etanercept and tocilizumab for management of a post-HSCT inflammatory disorder. Later re-evaluation of the lung biopsy showed rare CMV positive cells of unclear significance in the setting of ongoing CMV viremia.

The CMV viral load continued to improve on combination therapy with maribavir and foscarnet (to 94 IU/ml). Unfortunately, the patient continued to have complications of ARDS, prompting redirection of cares to comfort measures and the patient passed away.

#### Case discussion

Refractory or resistant CMV infection or disease occurs in cases where the CMV viral load continues to rise and/or symptoms of CMV disease fail to improve despite appropriate antiviral therapy for 2 weeks or more (7).

Refractory CMV infection is defined as a CMV viral load increasing by 1 log or more, or fails to decline by 1 log, after 2 weeks of appropriately dosed antiviral therapy. Probable refractory infection is considered if the viral load increases by <1 log after at least 2 weeks of appropriately dosed antiviral therapy. Refractory CMV disease occurs when symptoms are persistent despite at least 2 weeks of appropriate treatment. In cases of refractory infection, one must reassess status of immune suppression, confirm appropriate antiviral drug dosing and consider genotypic resistance testing. If resistance is present, drug therapy should be tailored to susceptible medications (Figure 1). Individual mutations can confer low- or high-level resistance, and multiple mutations can be additive, leading to high-levels of resistance (85).

As reported previously, maribavir is approved for treatment of refractory/resistant CMV infection or disease in patients 12 years of age and older. There is no dosing information available for children under 12 years of age. As this patient had multi-drug resistant CMV with few remaining therapy options and was clinically worsening on dual therapy with ganciclovir and foscarnet, we proceeded with full-dose therapy with maribavir in combination with continued foscarnet. This therapy did appear to have some effect, with decreasing viral load within 2 weeks of starting maribavir.

In this case, the patient also briefly received letermovir in the setting of ARDS and concern for CMV pneumonia. As noted previously, letermovir is not FDA approved for CMV prophylaxis or treatment in children, though is used off-label at some pediatric centers for prophylaxis and in select cases reported as salvage therapy (39–42, 86–88). With the lack of treatment options in resistant CMV infection, the favorable side effect profile of letermovir (including less bone marrow toxicity) and lack of cross-resistance with other antivirals (due to different therapeutic target sites), interest in the use of letermovir as salvage therapy has grown. As noted above, while some studies have shown a potential benefit with letermovir monotherapy or combination antiviral therapy in refractory or resistant CMV infection, resistance can develop quickly (66, 86).

Antiviral therapy is the mainstay of therapy for CMV infection and disease post-HSCT, though several adjunctive therapies are available. Adjunctive treatments are largely of questionable benefit, particularly in pediatrics. This patient received adjunctive treatment with leflunomide, artesunate and CMVIG. Leflunomide is an immunosuppressive drug typically used to treat autoimmune conditions or solid-organ transplant rejection (89). Leflunomide has also been found to have novel anti-CMV activity (either by inhibition of pyrimidine synthesis or inhibition of tyrosine kinase activity) and potential use in treatment of refractory/resistant CMV infection and disease (89–91). Artesunate, an anti-malarial medication, is thought to have antiviral activity *via* inhibition of CMV replication by interference with host cell kinase signaling systems (92). Studies on use of artesunate in resistant CMV infections have shown mixed results, with most success noted in mild CMV infection without organ involvement, though failure to prevent development of disease in some patients (93–95). The benefit of CMVIG as salvage or adjunctive therapy is also questionable, though is generally well tolerated.

### Case presentation 3: CMV pre-engraftment

A 3-year-old girl with acute lymphoblastic leukemia (ALL) and stage 4 neuroblastoma was admitted for allogeneic HSCT from a MUD (CMV D+/R+, EBV D−/R−). She received myeloablative conditioning with total body irradiation, cyclophosphamide 60 mg/kg, and etoposide 1,500 mg/m^2^. Within 1 week following HSCT, she developed CMV reactivation with low-level viremia (<100 IU/ml). Viremia was initially monitored without treatment, though with rapid rise of nearly one-log within 4 days (up to 424 IU/ml), she was started on foscarnet 60 mg/kg/dose q12 h. Foscarnet was continued for nearly 2 weeks, though the patient was transitioned to ganciclovir 5 mg/kg q12 h when the viral load continued to rise, due to concerns for resistance. She was noted to have diarrhea and rising ALT, concerning for CMV enteritis. An endoscopy was performed, and pathology demonstrated a single inclusion of a normal-sized nucleus with no CMV-type cytomegalic changes; this finding was felt to be of questionable clinical significance. In the setting of concern for probable CMV gastrointestinal disease, she was started on adjunctive CMVIG once weekly. The patient continued to have fevers, diarrhea, and elevated liver enzymes, prompting extensive work-up including unrevealing CT chest/abdomen and stool testing positive for *C. difficile*. The patient was started on PO vancomycin for *C. difficile*. Additional evaluation revealed adenovirus viremia (45,720 copies/ml of plasma) providing an alternate explanation for colitis and hepatitis. Cidofovir 5 mg/kg weekly was subsequently added (in addition to ganciclovir) to the antiviral regimen to provide treatment for adenovirus.

The patient responded to ganciclovir and was transitioned to valganciclovir. However, after a little over a month of ganciclovir/valganciclovir therapy, the CMV viral load rose substantially (up to 19,500 IU/ml). Throughout her treatment course, the patient had continued to have profound lymphopenia (<0.2 × 10^9^/L), which likely hindered her ability to mount an appropriate response to concurrent viral infections. Therefore, she was transferred to a study center for treatment with CMV- and adenovirus-specific CTLs. Letermovir 240 mg daily was also added for salvage therapy for approximately 1 week, later discontinued due to lack of evidence of benefit and to preserve letermovir for future prophylactic use.

CMV resistance testing was performed and demonstrated resistance to ganciclovir and cidofovir *via* A594V and T503I mutations, respectively. Ganciclovir was stopped and foscarnet 60 mg/kg q8 h was restarted. Cidofovir was continued for management of adenovirus viremia.

The patient underwent ophthalmologic exam shortly after diagnosis of CMV viremia that demonstrated no evidence of retinitis. However, approximately 1 month later, she was noted to have findings concerning for bilateral CMV retinitis, including white fibrotic lesions and white-centered intraretinal hemorrhages as well as a possible juxtafoveal lesion that was felt to be potentially vision-threatening. Despite this, the patient did not have any vision changes. A CMV PCR from the intravitreal fluid was negative. With concern for threatened vision and findings consistent with CMV retinitis, intravitreal foscarnet dose of 2,400 mcg was administered once at the time of intravitreal aspiration. Eye examinations were continued once weekly and demonstrated steady improvement. It was ultimately determined that ophthalmologic exam abnormalities might have been secondary to CMV retinitis or changes secondary to blood dyscrasia.

In the setting of ongoing lymphopenia and concern for graft failure, the patient ultimately received a second CTL infusion. She also received a peripheral blood stem cell boost with 4.86 × 10^6^ CD34 cells/kg from her original HSCT donor. Foscarnet was discontinued when the CMV quantitative PCR was undetected twice, measured 1 week apart, and she was transitioned to letermovir 240 mg PO daily for secondary CMV prophylaxis.

#### Case discussion

This case illustrates several principles in management of CMV infection and disease, including the diagnosis of CMV gastrointestinal (GI) disease, CMV retinitis monitoring and treatment, and adjunctive therapies.

Though not ultimately found to be the cause of this patient's diarrhea and transaminitis, CMV gastrointestinal disease is a well-known manifestation of CMV disease (81). CMV can affect the entire gastrointestinal tract (e.g., esophagitis, colitis). Clinical manifestations include abdominal pain, nausea, vomiting, diarrhea, GI bleeding and fever (96). Diagnosis is made based on the presence of upper and/or lower GI symptoms and CMV documented in tissue by histopathology, virus isolation, culture, immunohistochemistry, or DNA hybridization. Probable diagnosis is considered if the above are present, with proven or definite disease defined as presence of the above plus macroscopic mucosal lesions (7). It is important to note that CMV GI disease can present similarly to or occur concurrently with other conditions that can cause diarrhea, including intestinal GVHD, parenteral tube feedings, or other viral infections such as adenovirus. Therefore, one must have a high level of suspicion and pursue endoscopic evaluation with biopsies in patients with recent HSCT (especially within the first 100 days post-transplant) and abdominal symptoms.

Our patient was also evaluated for CMV retinitis, a potentially vision-threatening involvement of the eye. Early stage CMV retinitis is often asymptomatic, particularly in young children who may be unable to report or describe their symptoms (97). Even in the absence of symptoms, all HSCT patients with CMV viremia who are unable to clearly articulate visual symptoms should undergo thorough evaluation by an experienced ophthalmologist; this may require sedation in some children. Diagnosis of CMV retinitis is based on ophthalmologic examination alone in the majority of cases, with positive intravitreal CMV PCR considered as supportive of the diagnosis, especially in the presence of atypical ophthalmologic exam findings (7). Ophthalmologic findings consistent with CMV retinitis include areas of white/pale necrotic retina and focal areas of hemorrhage spreading centrifugally along vascular arcades (98). Treatment includes systemic antiviral therapy and/or intravitreal injections of antivirals (99–101).

As noted in case 2, adjunctive therapies including letermovir and CMVIG are of questionable benefit, particularly in the pediatric population.

### Case presentation 4: CMV during treatment for GVHD

A 12-year-old boy with chronic myeloid leukemia (CML) was admitted for allogeneic HSCT from a matched sibling donor (CMV D−/R+, EBV D+/R+). He received conditioning with busulfan, cyclophosphamide. He received daily tacrolimus and methotrexate on days +1, +3, +6 and +11 for GVHD prophylaxis. He tolerated HSCT well and engrafted on day +21. Due to his high-risk CMV status, he received letermovir 480 mg PO daily until day +100 with undetected weekly CMV blood PCR.

Approximately 3 months post-engraftment, the patient presented to the transplant clinic with a generalized rash, conjunctivitis, photophobia, and mouth sores. A skin biopsy was obtained which showed interface vacuolar dermatitis, focal subepidermal blisters and mixed dermal inflammation with few eosinophils, consistent with grade III GVHD. CMV stain of the skin biopsy was negative. He was treated with light therapy as well as prednisone 30 mg PO BID with improvement and subsequent slow steroid wean.

Five months post-engraftment, the patient was readmitted with chronic cough, progressively increasing shortness of breath and exercise intolerance. He was found to have low oxygen saturations in the mid-80s on room-air. A CT chest demonstrated multifocal ground-glass opacities bilaterally, predominantly in a peribronchial vascular distribution, scattered subpleural ground-glass opacities and mild cystic bronchiectasis. A bronchoscopy with BAL showed thick cloudy secretions in multiple segments with no mucosal edema and negative infectious evaluation, including negative bacterial, fungal, and mycobacterial cultures, negative *P. jirovecii* PCR and negative CMV PCR. With negative infectious work-up, the patient was diagnosed with pulmonary GVHD and started on 5 mg ruxolitinib PO daily.

Due to the risk of reactivation of CMV and EBV on ruxolitinib, CMV and EBV quantitative PCRs were monitored once weekly. Approximately 3 weeks after starting ruxolitinib, the patient developed CMV viremia up to 5,000 IU/ml. He was started on ganciclovir 5 mg/kg/dose q12 h with a rapid decline in CMV viral load. After 4 weeks of induction therapy with ganciclovir, the patient had 2 consecutive undetected CMV PCRs and he was transitioned to ganciclovir 5 mg/kg/dose q24 h followed by valganciclovir for maintenance while receiving treatment for GVHD.

#### Case discussion

This case demonstrates the importance of CMV monitoring, prophylaxis, and treatment during treatment for GVHD. Immune suppression given for treatment of GVHD increases the risk of several infections, including reactivation of herpesviruses, other viral illnesses, fungal infections and bacterial infections (5). Some GVHD management strategies may increase risk of CMV reactivation compared to others. Specifically, post-transplant cyclophosphamide has been associated with increased incidence of CMV infection in both haploidentical and matched HSCT (102, 103).

During treatment for GVHD, patients should have serial monitoring for reactivation of herpesviruses, including both CMV and EBV. Antiviral induction therapy should be initiated with detection of CMV viremia and continued until CMV viremia has resolved. Following resolution of viremia, regular CMV monitoring with pre-emptive antiviral therapy vs. secondary prophylaxis should be continued until the patient has completed therapy for GVHD and risk factors for CMV reactivation are no longer present (104).

### Case presentation 5: Late-phase CMV

A 16-year-old boy with refractory acute myelogenous leukemia (AML) underwent a haploidentical allogeneic HSCT (CMV D+/R+, EBV D+/R+). He had previously received two cycles of FLAG-IDA chemotherapy. He received conditioning with fludarabine 25 mg/m^2^ for 3 days and total body irradiation 150 cGy BID for 4 days. Though he initially appropriately engrafted, he subsequently developed lymphopenia as low as 0.44 × 10^9^/L (normal 1.0–3.2 × 10^9^/L). His post-transplant course was complicated by peripheral demyelinating and axonal sensorimotor neuropathy (requiring plasma exchange and rituximab), aspiration pneumonia, ventilator-associated pneumonia, central line-associated bloodstream infection, and pulmonary aspergillosis. He continued to receive prophylactic antivirals, letermovir and acyclovir, which were begun in the immediate peri-transplant period, until he demonstrated appropriate lymphocyte recovery.

At that time, immune competence studies were performed to determine if ongoing antiviral prophylaxis was required. These studies were relatively reassuring with a CMV immune competence assay consistent with effective immunologic response, normal lymphocyte proliferation to mitogens and moderately decreased lymphocyte proliferation to antigens. With this reassuring evaluation, consistent improvement of ALC to >1.0 × 10^9^/L and normal CD4 count at 600 cells/mcl (normal 497–2,267 cells/mcl), both antivirals were stopped approximately 6 months after engraftment. CMV PCRs were monitored once weekly for 4 weeks after stopping letermovir. One month after stopping CMV prophylaxis, the patient was noted to have a CMV viral load of 454 IU/ml, which increased to 826 IU/ml 2 days later with concurrent ALC of 2.0 × 10^9^/L. He remained an outpatient and clinically stable. Valganciclovir was started with rapid improvement in viral load to 43 IU/ml.

Given the history of long-term antiviral therapy, resistance testing was sent and revealed a L501F mutation in UL54, conferring predicted resistance to ganciclovir and cidofovir. However, given his rapid response to valganciclovir, this therapy was continued. The patient completed a total of 4 weeks of therapy with valganciclovir, having two undetected quantitative CMV PCRs documented prior to completing therapy. The patient returned to his home country during this time and recommendations were provided to administer secondary CMV antiviral prophylaxis and repeat immunologic testing.

#### Case discussion

This case demonstrates the ongoing risk of CMV reactivation in the late-phase (>100 days) following HSCT. Risk factors for late-phase reactivation include allogeneic HSCT (most notably MUD or T-cell depleted HSCT), chronic GVHD, steroid use, low lymphocyte counts (particularly low CD4), and delay in development of high-avidity anti-CMV antibody (105).

CMV immune competence assays, which quantitatively and qualitatively measure T-cells against CMV antigens, are used as a means of evaluating immune reconstitution following HSCT or solid-organ transplant. Research indicates that recovery of CMV-specific CD4+ and CD8+ T-cells is important in controlling CMV disease after HSCT (106).

This patient experienced CMV reactivation following T-cell reconstitution and demonstration of CD8 immune competence. It should be noted that this patient did not develop CMV disease and, despite predicted resistance to ganciclovir, this patient responded to a rather short course of therapy with valganciclovir. Both findings are likely secondary to immune reconstitution, improving the patient's ability to manage CMV reactivation without multiple or prolonged interventions.

## Discussion

Pediatric patients receiving HSCTs are at high risk of infectious complications from bacterial, fungal, parasitic, and viral pathogens. Among viruses, CMV is an important cause of illness in these patients, including life or vision-threatening disease. CMV must be considered at pre-transplant evaluations, at the time of transplant and in the early and late-phases post-transplant.

Prior to transplant, providers should ascertain donor and recipient CMV serostatus and consider the planned conditioning regimen and HSCT source to determine the ultimate risk of CMV infection and disease in each individual patient. CMV prophylaxis should be administered in patients at high risk for CMV infection and disease, or pre-emptive monitoring enacted to ensure early identification of viremia. As illustrated by the cases in this review, treatment of CMV infection can be complicated, particularly in HSCT patients, in whom T-cell recovery may be delayed, and considering the high incidence of myelosuppression with antiviral agents. Adjunctive therapies are available, though often have limited data support, particularly in the pediatric population.

Preventing and managing CMV in pediatric HSCT patients is a team effort with experts in stem cell transplant, infectious diseases, and pharmacy involvement. This review serves as a reference to manage these patients, including some of the most complex and difficult scenarios as illustrated by the cases presented in this report.
